# Learning deep architectures for the interpretation of first-trimester fetal echocardiography (LIFE) - a study protocol for developing an automated intelligent decision support system for early fetal echocardiography

**DOI:** 10.1186/s12884-022-05204-x

**Published:** 2023-01-11

**Authors:** Anda Ungureanu, Andreea-Sorina Marcu, Ciprian Laurentiu Patru, Dan Ruican, Rodica Nagy, Ruxandra Stoean, Catalin Stoean, Dominic Gabriel Iliescu

**Affiliations:** 1 Department of Paediatric Cardiology, University Emergency County Hospital Craiova, Tabaci, no.1, 200642 Craiova, Romania; 2 Department of Obstetrics and Gynecology, University of Medicine and Pharmacy Craiova, Petru Rares, no. 2, 200412 Craiova, Romania; 3 Department of Obstetrics and Gynecology, University Emergency County Hospital Craiova, Romania Tabaci, no.1, 200642 Craiova, Romania; 4 MEDGIN / GINECHO Clinic, 1 Mai, no. 29, 200333 Craiova, Romania; 5 Romanian Institute of Science and Technology, Virgil Fulicea, no. 3, 400022 Cluj Napoca, Romania; 6 Department of Computer Science, University of Craiova, A.I. Cuza, 13, 200585 Craiova, Romania

**Keywords:** Artificial intelligence, Decision support systems, Echocardiography, Fetal cardiology

## Abstract

**Background:**

Congenital Heart Disease represents the most frequent fetal malformation. The lack of prenatal identification of congenital heart defects can have adverse consequences for the neonate, while a correct prenatal diagnosis of specific cardiac anomalies improves neonatal care neurologic and surgery outcomes. Sonographers perform prenatal diagnosis manually during the first or second-trimester scan, but the reported detection rates are low. This project’s primary objective is to develop an Intelligent Decision Support System that uses two-dimensional video files of cardiac sweeps obtained during the standard first-trimester fetal echocardiography (FE) to signal the presence/absence of previously learned key features.

**Methods:**

The cross-sectional study will be divided into a training part of the machine learning approaches and the testing phase on previously unseen frames and eventually on actual video scans.

Pregnant women in their 12–13 + 6 weeks of gestation admitted for routine first-trimester anomaly scan will be consecutively included in a two-year study, depending on the availability of the experienced sonographers in early fetal cardiac imaging involved in this research.

The Data Science / IT department (DSIT) will process the key planes identified by the sonographers in the two- dimensional heart cine loop sweeps**:** four-chamber view, left and right ventricular outflow tracts, three vessels, and trachea view. The frames will be grouped into the classes representing the plane views, and then different state-of-the- art deep-learning (DL) pre-trained algorithms will be tested on the data set. The sonographers will validate all the intermediary findings at the frame level and the meaningfulness of the video labeling.

**Discussion:**

FE is feasible and efficient during the first trimester. Still, the continuous training process is impaired by the lack of specialists or their limited availability. Therefore, in our study design, the sonographer benefits from a second opinion provided by the developed software, which may be very helpful, especially if a more experienced colleague is unavailable. In addition, the software may be implemented on the ultrasound device so that the process could take place during the live examination.

**Trial registration:**

The study is registered under the name „Learning deep architectures for the Interpretation of Fetal Echocardiography (LIFE)”, project number 408PED/2020, project code PN-III-P2–2.1-PED-2019. Trial registration: ClinicalTrials.gov, unique identifying number NCT05090306, date of registration 30.10.2020.

## Background

Congenital Heart Disease (CHD) is the most encountered fetal malformation worldwide. The incidence of congenital heart disease appears to be about 1 per 100 live-born infants and is even higher in infants who die before term [1].

Fetal echocardiography (FE) has evolved from just the description of the anatomical abnormalities of the heart toward quantitative assessment of its function, dimension, and shape [2]. Presently, FE is performed manually by the sonographer during the first or second-trimester investigation. However, cardiac malformations were prenatally detected in only half of the babies undergoing surgery within the first year of life [3].explaining the need for an improvement of the fetal cardiac assessment. A correct FE interpretation is critical because it allows a detailed discussion regarding the prognosis with the parents (i.e., procedural risks, long-term mortality, morbidity, and, ultimately, quality of life). Also, it has a great potential to optimize the postnatal outcome. The lack of prenatal identification of congenital heart defects can adversely affect the neonate [4–6], while a correct prenatal diagnosis of specific cardiac anomalies improves neonatal care [7–11], neurologic [12] and surgery outcome [5, 13, 14].

Many studies showed significant discrepancies between the pre-and postnatal diagnosis of the CHD following classical FE [15, 16]. The reasons for the suboptimal performance of FE are related to the inherent limitations of ultrasound technique: the examination is experience-dependent and patient-dependent, therefore the competence and experience of the operator [17] and fetal-maternal conditions [18] (fetal positioning and size, maternal lower abdominal surgery, obesity [19], fibroids [20], placenta interposition or contractions), result in lacking consistency and reproducibility of the evaluation. For an adequate standardized examination of the fetal heart, the obstetric sonographers must achieve special competence and experience [21–23]. Also, fatigue and time pressure impact busy prenatal diagnosis units. Performing FE in the first trimester is challenging due to the small size of the heart [24], frequent unfavorable fetal position and involuntary movements [2], and the lack of expertise in early fetal echocardiography of some sonographers.

Previous studies proposed four-dimensional Spatio-temporal image correlation (4D STIC) to provide an audit/diagnosis for early pregnancy FE, as experts in local or telemedicine settings may later evaluate 4D STIC information [25–28]. However, important limitations hamper this approach: the lack of available high-tech equipment and experts for offline / telemedicine evaluations and experience to acquire good quality STIC volumes in general settings. Considering that these obstacles would be overcome, the time required for diagnosis is challenging to optimize. On the other hand, the cardiac sweep is always performed during general practice to evaluate cardiac normality by adequately visualizing cardiac anatomy key-planes. The first trimester FE involves some particularities due to the small dimensions and poor grey-scale visualization of the fetal heart structures; thus, color Doppler offers most information at this gestational age [29, 30].

Intelligent Decision Support Systems (ISs) are frameworks that can gather and analyze data, communicate with other systems, learn from experience, and adapt according to new cases. Technically speaking, ISs are advanced machines that observe and respond to the environment that they have been exposed to using Artificial Intelligence (AI) [31]. This project aims to foster a cross-fertilization of FE and ISs, which will provide an enormous potential in developing new fundamental theories and practical methods that rise above the boundaries of the disciplines involved and lead to new impactful methods that assist medical practice and discovery.

Several projects use new technologies to improve the primary acquired images, help extract measurements, or aid in diagnosing cardiac abnormalities, to improve the chances for optimal assessment of the fetal heart. These current approaches use Deep-Learning (DL) to diagnose CHD with FE [32–35] only for second-trimester screening. The innovation and originality of this project are not only to address first trimester FE but also to design and develop a specialized IS platform that embeds an ensemble of techniques aimed to analyze standard ultrasound acquisitions. Thus, the study will use information retrieved during standard evaluation, and there is no need for additional investigation (such as 4D acquisitions) for software development and implementation. This is important because the 4D STC technique is highly dependent on the sonographer's experience, the examination settings (maternal characteristics, fetal quintessence) and involves high equipment costs and more time necessary to complete the heart evaluation.

## Methods/design

### Aim

The aim of this project is the development of an IS that uses two-dimensional (2D) video files of cardiac sweeps obtained during the usual manually performed FE in the first trimester to signal the presence/absence of previously learned key features up to characteristic semantic segmentation when the structures are more developed anatomically. Also, the software will provide a counseling aid for inexperienced or newly trained sonographers and is expected to improve their expertise; the necessity to achieve proper images of all key features of the cardiac sweep has the potential to optimize image acquisition protocols and to ensure proper quality of the scan, which will result in a better evaluation of cardiac normality; raise the awareness and interest of academia to use the software during training of young sonographers; reduce the rate of diagnosis discrepancies between the first-trimester echocardiography and second trimester and postnatal cardiac assessment.

### Study design and setting

The study to be performed is a cross-sectional study divided into two separated parts (the training part of the machine learning approaches within the proposed framework and the testing phase on previously unseen frames and eventually on actual video scans) that will take place in two screening and referral prenatal diagnosis units: MEDGIN/GINECHO Clinic and University Emergency County Hospital Craiova (a tertiary maternity hospital).

### Participants characteristics

We conducted a previous pilot study [36] during the development of this research question, where we noted a good acceptability of the method and most of the women had little difficulty deciding whether or not to have the scan protocol. This high acceptability was expected, as no supplementary US investigation was required.

All pregnant women in their first trimester are considered eligible for the study. They will be consecutively included in the study, depending on the availability of the US operators involved in this research. We will try to attract a large team of collaborators to investigate as many eligible cases as possible. The pregnancies with congenital heart disease will be excluded from the primary analysis.

Pregnant women will be admitted for their routine first trimester genetic and anomaly scan at 12-13 weeks of pregnancy. Gestational age will be determined based on the last menstrual period, confirmed with the first biometry dating scan.

### Procedures

#### Recruitment

The recruitment process of the eligible patients starts during their regular consultation when the sonographer verbally informs the women about the research and invites them to participate in the study. If the patients are interested in being part of the study, the sonographers will provide information regarding the nature and purpose. All patients involved will be asked to provide written consent in the presence of their healthcare professional during their consultations. All collected information will be treated as confidential.This study will recruit pregnant women in their first trimester from 2021-to 2022.

#### Interventions

All pregnant women that meet the inclusion criteria will be examined by trained sonographers with at least three years of experience with fetal cardiac imaging.

#### I. Data set

##### Ultrasound examination

Women recruited to participate in the study will undergo a fetal echocardiogram between 12-13+6 weeks of gestation. Appropriately competent practitioners will perform fetal scans. The ultrasound machines used are General Electrics

Voluson E10, E8 and E6, and Phillips Epiq Elite, with dedicated software for fetal heart assessment, equipped with convex transabdominal probes (frequency: 3-7MHz). Two-dimensional evaluation of each fetal heart includes a cine loop sweep obtained using Doppler or directional power Doppler techniques, from the four-chamber view plane by moving the transducer cranially towards the upper mediastinum, allowing the visualization of the following planes**:** four- chamber view, left and right ventricular outflow tracts, three vessels, and trachea view [37]. The acquisition time will be set for 5 seconds for each sweep. The cine loop sweeps will be stored on the hard drive of the ultrasound machine.

**Fig. 1:**
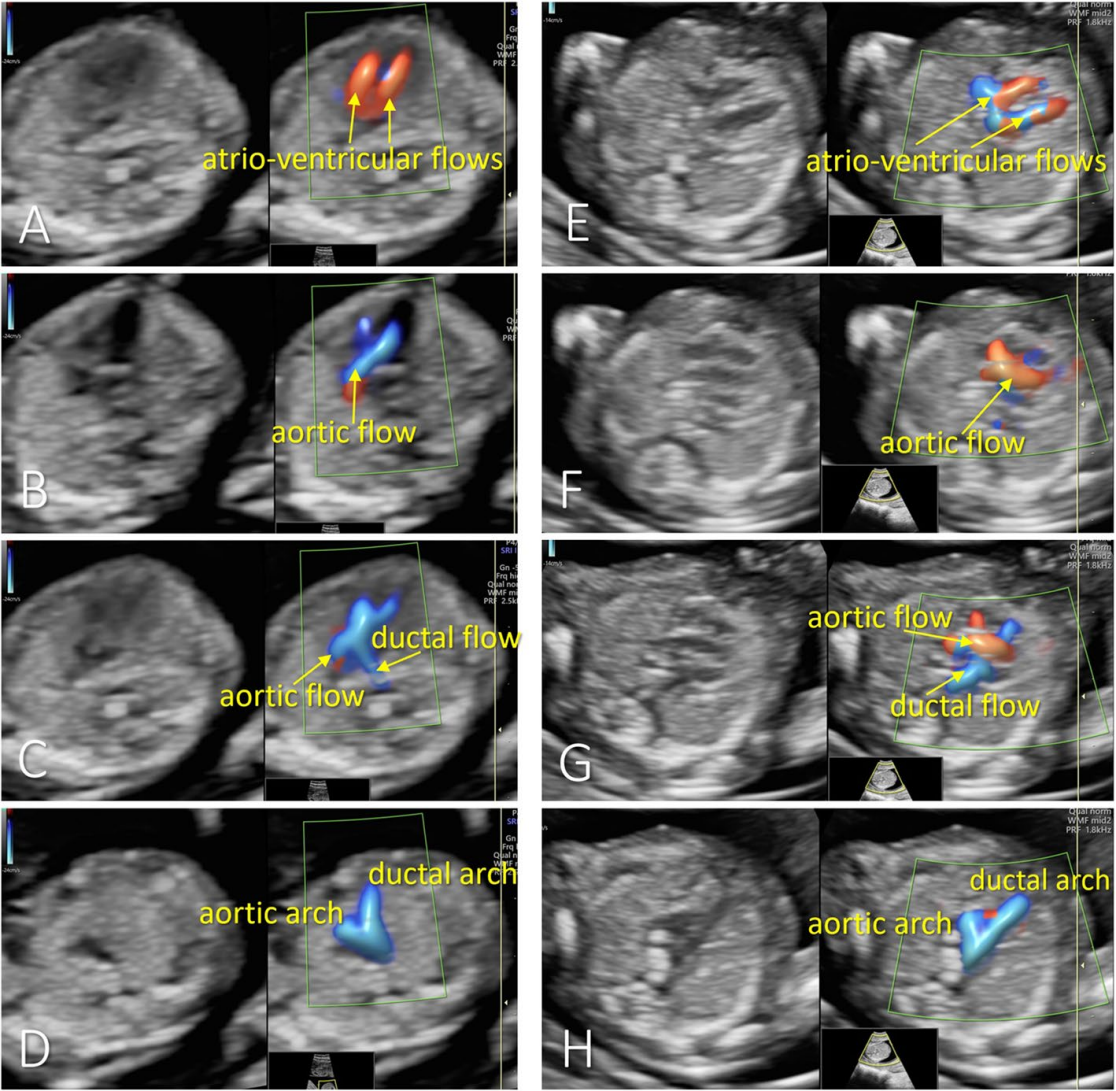
Examples of the four key-planes visualized during the first-trimester fetal heart sweep in apical view (**A-D**) and lateral view (**E-H**). From top to bottom (each on one row): the parallel atrioventricular flows in four-chamber view plane (**A,E**), the aortic flow in left ventricle outflow tract view (**B,F**), the X-sign – crossing of the arterial arches (**C,G**), and the V-sign, in the three-vessel plane (**D,H**)

**Fig. 2:**
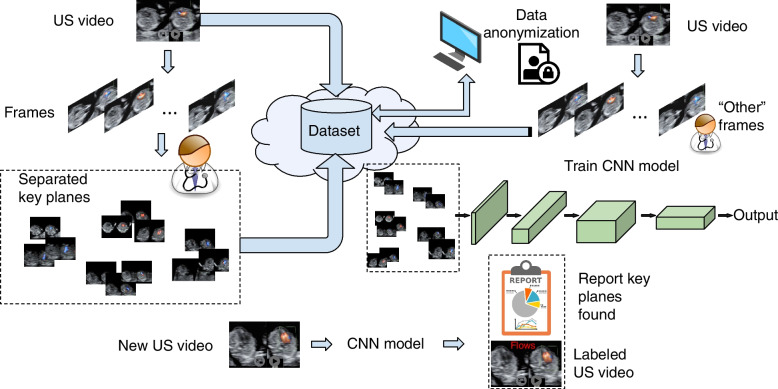
Workflow overview of the entire protocol. US videos are separated into frames, from which human experts assign the key planes to the four different categories. Data are anonymized, non-important frames are added as a distinct category of images, the model is trained on the obtained image data set and will then classify frames in new US videos and generate reports

The protocols for the first trimester examinations are presented in Figs. 1 and 2. An apical-oblique lateral or lateral incidence from the right part of the thorax is considered optimal for visualization of the key-planes essential for fetal cardiac evaluation [29, 30]. Therefore, the operator will focus on other segments of fetal anatomy until the fetus reaches the optimal incidence; the angle beam is narrowed, and high-definition zoom is applied until only the thorax becomes visible, occupying approximately 80% of the screen. The operator then turned on the color flow gate, and a minimum of one duplex video file (grayscale and color / directional power Doppler) will be stored in each patient. The cardiac sweep is aimed to contain the key-planes of heart assessment described below and presented in Fig. 1.

At the first-trimester anomaly scan, color Doppler is necessary because of the low discrimination of the heart structures in grey-scale mode. As presented in our previous research and literature, as well [38–40] we decided that color or directional power Doppler will provide the main features for evaluating the key views in the first trimester to confirm the normality of the blood flow in the corresponding structures. Appropriate video clips of fetal heart evaluation from apical and lateral incidence will be stored and included in the study prospectively. The following key-planes and color Doppler features will be searched in the FE video clips (Fig. 1):

1. Four-chamber view plane, showing atrioventricular equal flows and no significant flow between ventricles in color Doppler imaging.
2. Left ventricular outflow tract plane, showing the aortic flow with a typical origin, direction, and diameter in color Doppler imaging.
3. Crossing of the arterial arches, showing the intersection of the right ventricular outflow tract: (pulmonary and ductal flow), directed straight back towards the spine, with the aortic flow.
4. Three vessels view plane: color Doppler imaging shows the confluence of arterial arches with a similar diameter and normal flow direction in both arches.

##### Data set organization

Figure 2 presents a bird’s-eye view of the entire protocol. In order to create a deep learning model that would accurately identify the key-planes in a US video, a high number of samples for each of the four categories represented in Fig. 1 is necessary. Accordingly, all US videos that will serve for the training of the computational model will be split into separated frames. The physicians will select the appropriate images from these frames showing the cardiac key planes and distribute them to dedicated folders. These will be placed on a shared folder in cloud storage, where both physicians and computer scientists working on the task will have secured access. This way, multiple doctors can contribute with data, and, at the same time, the machine learning experts can access the continuously growing data set. Also, all the fetal echocardiograms from 2018 to the present will be reviewed to include them in the data set that will train the IS.

The video files will also be uploaded, besides the images separated in different folders based on the key planes classification. The uploaded videos will serve for automatic extraction of the frames that do not contain satisfactory key planes views.

#### II.Data preprocessing

All video files saved from the ultrasound (US) devices and the frames separated by the OB-GYN/Cardio (OBC) department into folders corresponding to the key planes are collected into the cloud. The Data Science / IT department (DSIT) will process the frames for obeying the anonymization regulations. This implies two essential aspects. One is obviously to erase the information that refers to the patients from the frame samples and the video files, as file and folder names. However, before deleting such information, unique strings will be assigned to each patient, such that, when separating the samples into sets for training, validating, and testing the model, all images that belong to the same patient will be placed in only one of the three sets. In the case that some samples from a patient would be placed in the training set and other samples from the same patient would be put in the validation (or test) set, the machine learning model would be biased since these samples would be more similar compared to those coming from different patients. Hence, before the anonymization takes place, unique codes will be generated for each patient in turn, and the code will appear on the names of the files.

Subsequently, the samples of the patients will be split into training, validation, and test sets, keeping the proportions of 60–20%-20%. These approximate proportions need to be kept for each class in turn. As mentioned above, images belonging to the same patient will be placed in the same set, be it training, validation or test. In addition, even if the samples come from a different video file but from the same patient, e.g., if the patient went to a consultation at another date, the samples will be placed in the same folder to avoid biasing the model.

**Fig. 3:**
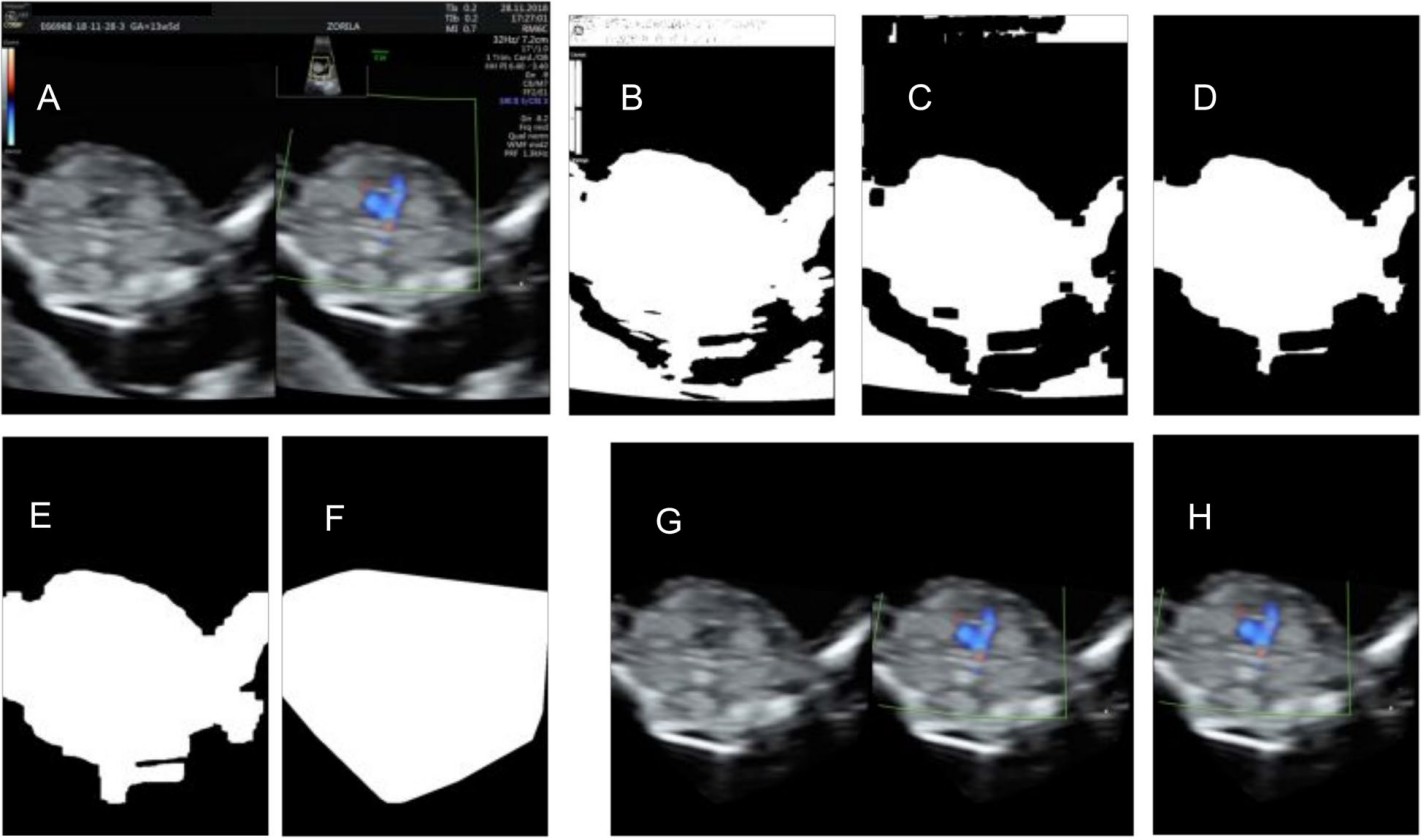
Examples in which background is removed from the initial image (**A**), intermediary images are obtained for one side (**B-F**), and either both views are kept (**G**), or the Doppler color side is used alone (**H**)

Several scenarios will be investigated concerning data preprocessing. The first option would be to keep the frames obtained after anonymization. A second approach would imply extracting from the frames both main ovals and removing the rest from the picture after creating a mask for the areas of interest (main ovals). This can be obtained by transforming the images to grayscale and later thresholding them. The noise that would remain in the foreground and background could be removed by applying several steps, as described next. The image could be split vertically in half, and each side could be dealt with similarly. Next, one half is discussed, but the other part is processed in the same way, and, at the end of the process, they can be merged into one image corresponding to the initial image. Thresholding is applied to obtain a mask where lighter pixels from the initial image (Fig. 3(A)) are transformed to white, and the rest remains black, as in Fig. 3(B). Next, erosion is applied for removing isolated pixels standing for noise, and a representation as in Fig. 3(C) is obtained; then, the largest contour is kept for removing the other detected parts that are not useful for classification, as illustrated in Fig. 3(D). Dilation is applied to take the white contour back to its size before erosion as in Fig. 3(E). Subsequently, a convex hull is applied for a less rugged contour that could deceive the model as illustrated in Fig. 3(F). Finally, after applying the obtained mask on the original frame image, a representation as in Fig. 3(G) would be obtained by merging the two sides. Only the Doppler oval could be kept in a third scenario, as in Fig. 3(H). Each of the three scenarios will be tried by training the deep learning model on each sample type. It is expected that the samples containing the Doppler color side alone (subfigure H in Fig. 3) would be faster to process, but they might not carry enough information. This latter could also be the case with the views in Fig. 3(G), or perhaps the manner of cropping might even influence the model’s decision. The scenarios will be tested by comparing the accuracy results on validation and test sets in all situations. Additional information could also be obtained from methods that provide visual explanations from convolutional neural networks, like Grad-CAM [41].

#### III. Frame Classification

As previously stated and illustrated in Fig. 2, the deep learning model needs another class to be learned, apart from the ones illustrated in Fig. 1. This class should not contain any key plane; hence we labeled it *other*, meaning it is unimportant (not informative). It will be appointed to make the machine recognize the key features from the sweeping, where the majority represents noise or transition between the views. They do not belong to any of the key planes, and the model should appropriately identify them as not informative. Contrarily, if the model were trained without the *other* class, for each frame that does not contain a particular plane, the algorithm would feel forced to label it with one of the classes of interest, even if that image does not represent anything of value for the detection.

Accordingly, the saved US video files will allow the automatic extraction of the frames that do not confidently present any of the key planes, i.e., the *other* samples. The images extracted from the video frames by the OB-GYN experts will be used for comparison with the frames from the video files, and, when the similarity is below a certain threshold, the frames will be considered distinct enough from the images of the key planes and will be saved to the *other* folder. The number of such non-important images can be very high, but it will be controlled by establishing an adequate number of samples, i.e., around 30% more samples than in the class with the most images. The higher the quantity of the images in the *other* class, the better the model will learn. If not enough samples are assigned to this class, images that do not contain key planes may be labeled as being from such classes. Hence, it is crucial to have a proper value for the number of items in class *other*, and this value will be empirically determined. After these transition samples are extracted, the human experts will again check the samples to ensure that key planes would not be included by mistake. DSIT will try different state-of-the-art DL pre-trained algorithms on the data set with the plane views. The training will take place only on images, not on video files at this stage. After training, the model that proved to be the most accurate on the validation set will be saved, and it will be included in an application that will be able to deal with video files.

#### IV. Experiments

All the recent DL entries will be tailored and tested on the current scenario of key view identification. Their prediction results will be analyzed compared to the ground truth marked by the OBC. The metrics concern accuracy, precision, recall, and F1-score. A hierarchy will be built based on statistical testing, and the best algorithm will represent the AI module for this scenario. Different architectures can be tried. Some have a relatively small number of layers, and they are faster as concerns running time. Others are very deep, meaning that they have a high number of layers hence they take longer to train but are potentially more accurate. It is essential to be fast in the testing stage for the final trained model used in practice, as the training model would not be later re-initiated. Although the more complex models still run slower in the testing phase than those with fewer layers, the differences are minimal. However, the current task needs to be thoroughly tested since it might be the case that shallow models also lead to accurate results and, if this is the case, they will be preferred. The accuracy-speed equilibrium will be considered when ranking the approaches since the system will finally perform on a video.

Once a new video is available in practice, the model chosen for the task will highlight each key feature and the probability/certainty. Additionally, the final application will present a graphical and numerical report showing the number of frames with key planes identified and the indication of the certitude it has for them. Finally, the OBC physicians will validate all the intermediary findings at frame level and the meaningfulness of the video labeling.

#### V. Sample size calculation

As opposed to classical statistics, for machine learning in general, but for deep learning tasks that involve image classification in particular, there is not a general pre hoc rule for calculating the sample size. The pre hoc model-based approaches rely on the features of the algorithms taken into consideration. We underline that the approaches to be used are non-linear, in fact they are from the deep learning family. The classification accuracy will continue to improve as more data is included in the training process, as opposed to standard machine learning algorithms, where the accuracy reaches a plateau when the data attains a certain threshold. The statistical heuristic methods often take into account the number of classes, the number of input features and the number of parameters of the model. However, considering that the number of parameters of a deep learning approach is usually of many millions and that for each distinct parameter there are usually tens of samples necessary, a statistical heuristic method becomes unusable. A rule of thumb in deep learning is to have 1000 samples per class, as used in the ImageNet dataset pioneered in the Large Scale Visual Recognition Challenge 2012. The number of samples is smaller than what a heuristic estimate would indicate, since transfer learning will also be employed and additionally the data set will be artificially enlarged via image augmentation. Accordingly, it is intended to have at least 6000 images (around 1000 per class) within our data set, which could be extracted from approximately 300 ultrasound video files. We intend to make a post hoc sampling estimation study by considering subsets of the data for training and observing the results on the same test set.

### Outcome measures

#### Primary outcome

The primary outcome of this study is an IS that can assist the early-stage sonographers in helping and training for accurate detection of the four first-trimester cardiac key-planes.

#### Secondary outcomes

- The increase in the rate of satisfactory evaluations of the heart key-planes in first trimester scans by inexperienced and newly trained sonographers.
- The optimization of the ultrasound clinical workflow using AI as an assistant for the FE practice through a time- efficient video labeling, i.e., a video frame processed in less than 20 milliseconds. Several options could be tried to achieve this, and the optimal decision will perhaps include all of them. We intend to vary the preprocessing options since some might be time-consuming. Another option refers to resizing the input images to a smaller dimension as long as the accuracy does not significantly decrease since they are faster to process when the size of the input data is reduced. Changing the deep learning model architecture to one with fewer layers would also reduce the amount of time for the processing of video frames.
- A reduced rate of diagnosis discrepancies between evaluators with different experience.

### Statistical methods

There will be several deep learning architectures trained on the obtained data set. In order to select the most prolific one and be assured that its results are not based on chance, the performance of the different models tried in terms of the considered metrics will be assessed under random sub-sampling cross-validation with multiple repetitions and the significance in the difference of the results will be checked via a Wilcoxon signed-rank test.

The performance of the computational models on videos taken by inexperienced sonographers will be statistically assessed compared to the results on scans where an expert made the examination. In order to achieve this, two sets of video scans will be completed, one containing video files made by experienced sonographers and the other made by inexperienced ones. The frames that the computational model labels will be extracted from the scans, and the experienced physicians will validate their predictions for both types of data. A t-test and a Mann-Whitney test for independent test samples will be used for verifying the differences in the results on the two sets.

## Discussion

### Study strengths

The study provides the first AI standardized approach to identifying fetal cardiac anomalies from fetal echocardiography (FE) sweeps in the first trimester.

The study aims to overcome the main limitations of the current approaches for automatizing the FE that target only the second-trimester scan and use Deep-Learning (DL) only to diagnose cardiac heart disease (CHD). In this respect, the techniques used for the automatized interpretation of cardiac ultrasound sweeps will range from timely key feature detection. In addition, the Intelligent Decision Support System (IS) will also be statistically validated at each step.

The study will use information retrieved from general practice during standard evaluation. Therefore, there is no need for additional investigation, such as 4D acquisitions for software development or implementation. Also, the 4D STC technique is highly dependent on the sonographer’s experience, the examination settings and involves significant equipment and additional time costs.

### Study limitations

A limited number of ultrasound devices types and techniques used to acquire the ultrasound sweeps may represent an impediment to large-scale implementation of the study results. The imaging of other ultrasound systems than the ones used to complete the study may differ significantly in the color format, and the performance of the software could be lower. We address this issue by using several color and directional power Doppler techniques and different generations of equipment in the study.

According to the study design, we will include in the study normal hearts; thus, the accuracy for detection of CHD cannot be evaluated from these data.

FE is feasible and efficient during the first trimester. Still, the continuous training process is impaired by the lack of specialists or their limited availability. Therefore, in our study design, the sonographer benefits from a second opinion provided by the developed software, which may be very helpful, especially if a more experienced colleague is unavailable. In addition, the software may be implemented on the ultrasound device so that the process could take place during the live examination.

Also, this software may help improve heart scanning skills because the operator will be stimulated to obtain satisfactory images regarding the key-planes of the cardiac sweep.

This study will provide insight into the feasibility of automatization of the 2D-FE. The aim is to provide a more early and accurate prenatal diagnosis of CHD to improve the treatment options for parents: pregnancy termination or birth in a specialized center. This will ultimately improve the couple’s satisfaction regarding the first-trimester anomaly scan performance, infant survival rates, and the quality of life of the newborns.

### Reporting of adverse events

Ultrasound in pregnancy is the most commonly used and powerful tool for diagnosing CHD. The general belief is that diagnostic ultrasound (DUS) does not pose any risk to the pregnant patient or her fetus. Ultrasonography is free of ionizing radiation, but during the examination, it is accompanied by energy deposition in the tissue [42], so there is the potential to initiate biological effects [43]. However, we will analyze the stored videos acquired during the standard protocol during our study, and no supplementary evaluation is required. When using color/power Doppler, the mechanical and thermal indices will be kept as low as possible (ALARA principle) according to safety guidelines [44–46].

In case of any adverse event reported by the patient or observed by the obstetrician, the health professional will act accordingly to ensure patient safety.

## Data Availability

The study results will be disseminated at national and international research conferences and as published articles in peer-reviewed journals. Also, the study will be implemented and reported in line with the STROBE statement. All the anonymized collected data will be processed and stored in the www.zenodo.org research depository. Access to the raw data can be obtained from Iliescu Dominic Gabriel, contact details, Iliescu.dominic@yahoo.com, phone no. 0040723888773.

## Abbreviations

FE: Fetal echocardiography
DSIT: The Data Science / IT department
DL: Deep-learning
CHD: Cardiac heart disease
IS: The Intelligent Decision Support System
4D STIC: Four-dimensional Spatio-temporal image correlation
AI: Artificial Intelligence
2D: Two-dimensional
US: Ultrasound
OBC: OB-GYN/Cardio
DUS: Diagnostic ultrasound
