# Correction: Learning deep architectures for the interpretation of first‑trimester fetal echocardiography (LIFE) ‑ a study protocol for developing an automated intelligent decision support system for early fetal echocardiography

**DOI:** 10.1186/s12884-023-05825-w

**Published:** 2023-07-05

**Authors:** Anda Ungureanu, Andreea‑Sorina Marcu, Ciprian Laurentiu Patru, Dan Ruican, Rodica Nagy, Ruxandra Stoean, Catalin Stoean, Dominic Gabriel Iliescu

**Affiliations:** 1 Department of Paediatric Cardiology, University Emergency County Hospital Craiova, Tabaci, no.1, Craiova, 200642 Romania; 2 Department of Obstetrics and Gynecology, University of Medicine and Pharmacy Craiova, Petru Rares, no. 2, Craiova, 200412 Romania; 3 Department of Obstetrics and Gynecology, University Emergency County Hospital Craiova, Romania Tabaci, no.1, Craiova, 200642 Romania; 4 MEDGIN / GINECHO Clinic, 1 Mai, no. 29, Craiova, 200333 Romania; 5 Romanian Institute of Science and Technology, Virgil Fulicea, no. 3, Cluj Napoca, 400022 Romania; 6 Department of Computer Science, University of Craiova, A.I. Cuza, 13, Craiova, 200585 Romania

**Correction: BMC Pregnancy Childbirth 23, 20 (2023)**

10.1186/s12884-022-05204-x

Following publication of the original article [1], the authors identified an error in the author names. The given name and family name were erroneously transposed.

The incorrect author names are: Ungureanu Anda, Marcu Andreea-Sorina, Patru Ciprian Laurentiu, Ruican Dan, Nagy Rodica, Stoean Ruxandra, Stoean Catalin, Iliescu Dominic Gabriel.

The correct author names are: Anda Ungureanu, Andreea-Sorina Marcu, Ciprian Laurentiu Patru, Dan Ruican, Rodica Nagy, Ruxandra Stoean, Catalin Stoean, Dominic Gabriel Iliescu.

The author group has been updated above and the original article [1] has been corrected.

## References

[1] Anda U, Andreea-Sorina M, Laurentiu PC (2023). Learning deep architectures for the interpretation of first-trimester fetal echocardiography (LIFE) - a study protocol for developing an automated intelligent decision support system for early fetal echocardiography. BMC Pregnancy Childbirth.

